# Designing Dual-State and Aggregation-Induced Emissive Luminogens from Lignocellulosic Biosourced Molecules

**DOI:** 10.3390/molecules29133135

**Published:** 2024-07-01

**Authors:** Maelys Bonnot, Nagham Ibrahim, Magali Allain, Pierre Frère

**Affiliations:** UNIV Angers, CNRS UMR 6200 MOLTECH-Anjou, 2 Boulevard Lavoisier, 49000 Angers, France; maelys.bonnot@univ-angers.fr (M.B.); nagham_ibrahim5@hotmail.com (N.I.);

**Keywords:** cyanostilbene derivatives, dual-state emission, bio-sourced materials, fluorescence emission

## Abstract

Utilizing lignocellulosic biosourced platforms, we synthesized novel cyanostilbene (CS) derivatives featuring the 3,4-dimethoxyphenyl moiety. These derivatives were investigated for their emission properties in both solution and solid states. The two simple CS derivatives exhibit very weak luminescence in solution but significant luminescence in the solid state, indicating distinct Aggregation-Induced Emission (AIE) characteristic. Furthermore, combining these two CS units, without conjugation and with quasi perpendicular orientation, results in a Dual-State Emission (DSE) fluorophore showing luminescence both in solution and solid states. X-ray crystallography studies on the solid-state compounds reveal a structure–emission relationship, demonstrating that the colour emission correlates with the conformations adopted by the molecules in the solid state, which influence the type of stacking.

## 1. Introduction

In the field of organic fluorophores, the design of molecular structures plays a pivotal role in achieving efficient emission, whether in dilute solutions or within aggregates, powders, and crystals [1,2]. Rigorous π-conjugated derivatives often exhibit high luminescence in diluted solutions, with emission color strongly influenced by molecular structural factors like π-extension and intramolecular donor–acceptor charge transfer. However, many of these molecules experience a significant reduction or even quenching of their emission as the concentration increases, leading to aggregate formation driven by non-radiative deactivation pathways such as cofacial π–π stacking and the presence of trap states. Beyond these conjugated molecules, often termed ACQ derivatives due to their aggregation-induced quenching behavior, a diverse array of purely organic compounds exhibits an opposing effect, characterized by solid-state luminescence enhancement (SLE) or Aggregation-Induced Emission (AIE) [3,4,5,6]. Lastly, a third category of luminescent derivatives, known as Dual-State Emission (DSE), possesses the unique attribute of luminescing both in solution and in the solid state [7,8,9,10,11,12].

While the majority of conjugated systems are synthesized using raw materials sourced from fossil resources, the incorporation of biomass-derived units into these systems is currently emerging in order to find ways to produce sustainable materials that have a lower environmental impact [13,14]. Biomass feedstocks, such as agricultural residues and wood chips, constitute an inexpensive renewable resource for commercial large-scale biorefineries, as these waste products are widely available and can sequester carbon. Among the different biosourced platforms [15], lignocellulosic biomass, mainly composed of cellulose, hemicellulose, and lignin [16], offers the advantage of remarkably short production cycles, often less than five years, and holds the potential to yield a diverse array of polyfunctional aromatic molecules, such as furaldehydes and hydroxybenzaldehydes [17] that can be highly relevant for the creation of specialized conjugated systems [18,19]. Furaldehyde derivatives, such as methylfurfural and hydroxymethylfurfural (HMF), are derived from cellulose [20], while lignin enables the extraction of phenol derivatives such as vanillin or analogs [21,22,23,24].

**Figure 1:**
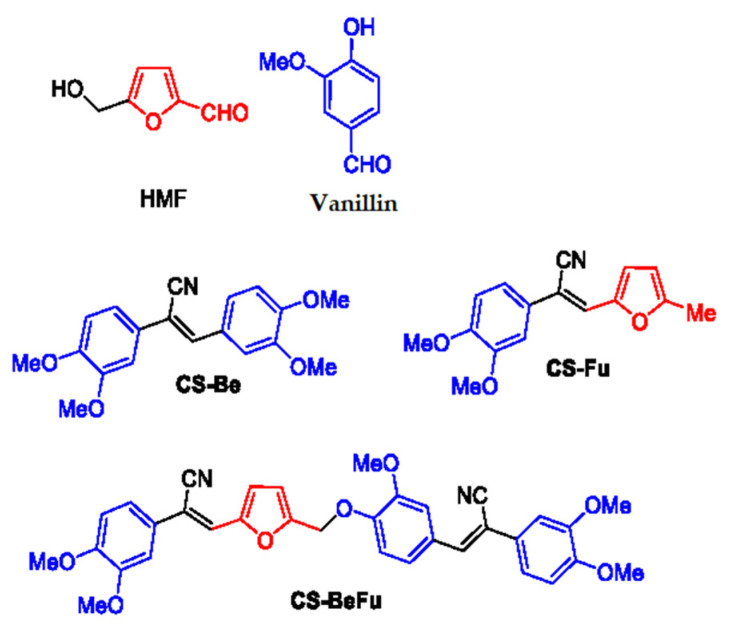
Biosourced molecules HMF and vanillin and structures of the synthesized cyanostilbene luminophores **CS-Be**, **CS-Fu**, and **CS-BeFu**.

In our ongoing projects that delve into harnessing biosourced molecules derived from plants and the development of compounds with extended π-conjugation for electronic properties and luminescence [25,26,27], we introduce here a novel series of cyanostilbene (CS) derivatives integrating 3,4-dimethoxyphenyl and furan units, capitalizing on biosourced raw materials such as vanillin and HMF (Figure 1) [28,29]. α-Cyanostilbene derivatives are highly attractive luminescent materials in the solid state with photophysical properties that are modulated both by the diversity and structural flexibility of the molecules and by the types of stacking of the molecules that generate the solid [28,29,30,31,32]. Most compounds of the CS family exhibit an AIE effect with much greater luminescence in the solid state than in solution. However, through strategic design modifications of CS derivatives, recent synthesis efforts have yielded several compounds that demonstrate varying levels of emission in solution [33,34,35,36,37,38,39]. We showcase how uncomplicated structural modifications empower us to craft compounds with remarkably diverse luminescent characteristics, spanning from low-emission systems to those exhibiting AIE or DSE behavior. Furthermore, the use of Knoevenagel condensation to generate cyanovinyl bonds, a sustainable and green reaction occurring under mild conditions without generating toxic byproducts, enables the exploration of luminescent material development within the framework of sustainable development.

## 2. Results and Discussion

### 2.1. Synthesis

**Scheme 1:**
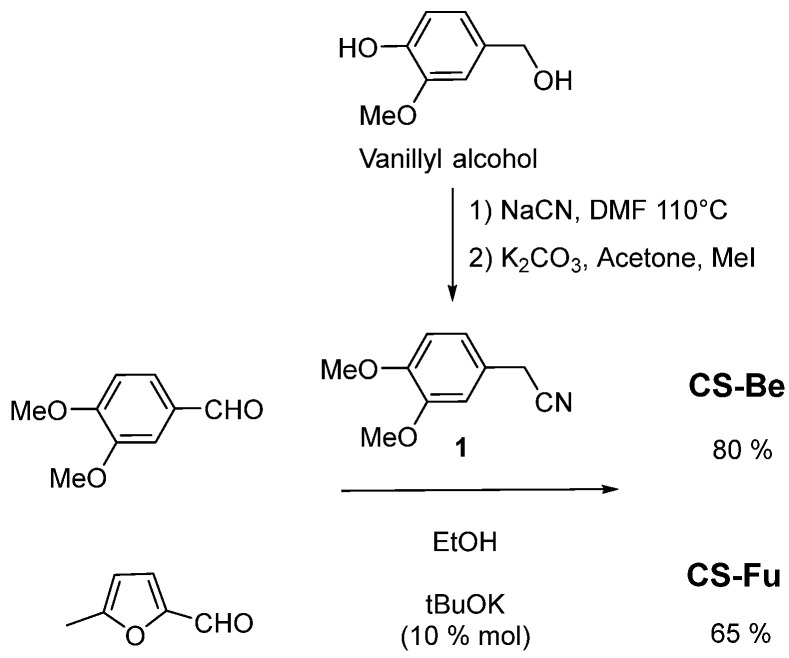
Knoevenagel condensation with the benzyl cyanide **1**.

All the compounds were synthesized in the last step by a Knoevenagel reaction involving the benzyl cyanide derivative **1**, easily obtained in two steps from vanillyl alcohol [40]. The Knoevenagel condensations for obtaining the compounds **CS-Be** and **CS-Fu** from the benzylcyanide **1** with the corresponding aldehydes 3,4-dimethoxybenzaldehyde and methylfurfural were easily carried out in ethanol at room temperature in the presence of a catalytic amount of ^t^BuOK (Scheme 1). The obtained precipitates were filtered and washed with ethanol to give **CS-Be** and **CS-Fu** in 85% and 65% yields, respectively.

**Scheme 2:**
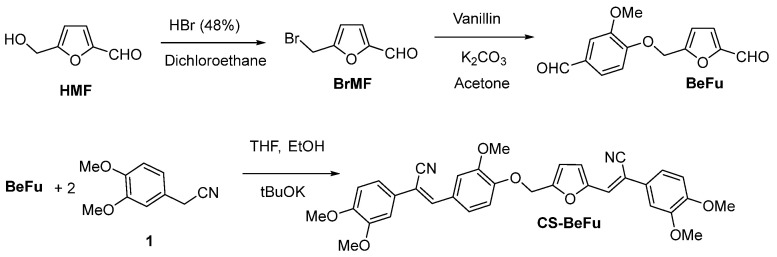
Synthesis of the dialdehyde **BeFu** and biscyanostilbene derivative **CS-BuFu**.

The dialdehyde derivative **BeFu** integrating both benzaldehyde and furaldehyde units was synthesized from vanillin and hydroxymethylfuraldehyde (**HMF**) (Scheme 2). Treatment of **HMF** solution in dichloroethane with concentrated bromhydric acid (48%) at room temperature leads to bromine derivative **BrMF** in 80% yield. The green oil obtained after the usual work up was directly engaged in a Williamson reaction with vanillin to afford the dialdehyde **BeFu** in 80% yield. Knoevenagel double condensation between the dialdehyde **BeFu** and a slight excess of three equivalents of benzylcyanide **1** was carried out in a mixture of tetrahydrofurane (THF) and ethanol (5/10 vv) at 50 °C in the presence of 10% mol of ^t^BuOK for 15 min. After cooling at 0 °C, a precipitate slowly appeared to form after filtration of the target molecule **CS-BeFu** in 55% yield.

The ^1^H NMR spectra of **CS-Be** and **CS-Fu** in CDCl_3_ are characterized by the presence of only one singlet corresponding to the signal of the vinylic proton. Moreover, an exact number of carbons was observed in the ^13^C NMR spectra of all the compounds. These NMR results provide sound evidence of only one configurational vinylene isomer in each solid sample. For **Cs-BeFu**, the ^1^H NMR spectrum in DMSO d_6_ (Figure S1 in the Supplementary Materials) shows that the two ethylenic protons have the same chemical shift at 7.91 ppm and all aromatic protons are well defined, as expected if each ethylenic bond exists in only one configuration. Single crystals of the three compounds, enabling characterization by X-ray diffraction, were obtained, thus allowing us to unequivocally confirm the Z-configurations of the cyanovinyl bonds in each structure (Figure S2 in the Supplementary Materials).

### 2.2. Electronic Properties

**Figure 2:**
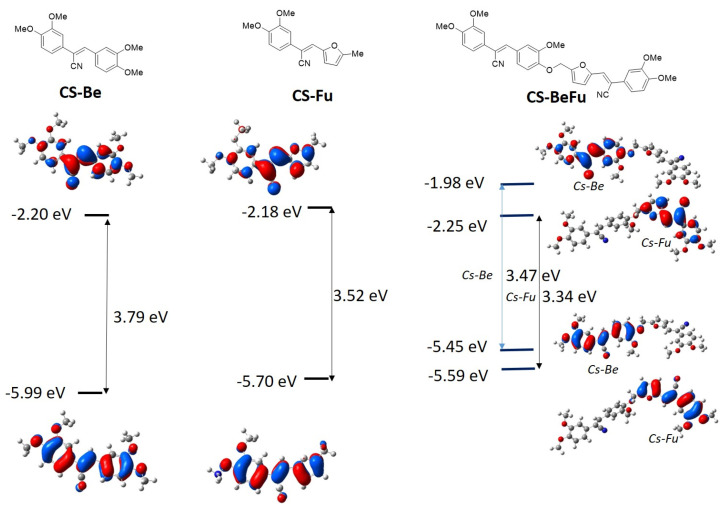
HOMO and LUMO frontier orbitals along with their corresponding enegy levels calculated by gas-phase DFT.

The electronic properties of the molecules were evaluated by theoretical calculation performed at the ab initio density functional level using the Gaussian 09 package. Becke’s three-parameter gradient-corrected function (B3LYP) with a polarized 6-311G(d,p) basis set was used for the geometrical optimization and for the HOMO and LUMO level determinations. The Z-configuration for the cyanovinyl units was used for the calculation for all the compounds. Figure 2 shows the representation of the HOMO and LUMO levels and indicates the energy levels of the three compounds. For both compounds **CS-Be** and **CS-Fu**, the HOMO exhibits delocalization over the entire conjugated system, while the LUMO is more localized on the central cyanovinyl bond. While the energy levels of the LUMO are very close for **CS-Be** and **CS-Fu**, the energy level of the HOMO for **CS-Fu** is destabilized compared to that of **CS-Be**, indicating a more electron-donating character of the methylfuran group than the 3,4-dimethoxyphenyl group. Consequently, the HOMO–LUMO gap is slightly lower for **CS-Fu** than for **CS-Be**. Geometrical optimization for the **CS-BeFu** molecule reveals that the two CS units named *Cs-Fu* and *Cs-Be* lie in two nearly perpendicular planes. Energy calculations lead to very close HOMO and HOMO-1 levels, with HOMO-1 localized on the *Cs-Fu* part and HOMO on *Cs-Be*. Similarly, the close LUMO and LUMO+1 levels show localization on the *Cs-Fu* and *Cs-Be* parts, respectively. Overall, the respective gaps for each of the two parts, *Cs-Fu* (HOMO-1–LUMO) and *Cs-Be* (HOMO–LUMO+1), are lower than those calculated for the **CS-Fu** and **CS-Be** molecules.

### 2.3. Optical Data

The UV–Vis absorption and fluorescence were performed in dilute (10^−5^ M) cyclohexane solution, and then the fluorescence was also measured in the solid state. For the fluorescence, the quantum yields Φ correspond to the absolute values obtained from an integrated sphere. All the data are gathered in Table 1.

#### 2.3.1. Optical Properties in Solution

**Figure 3:**
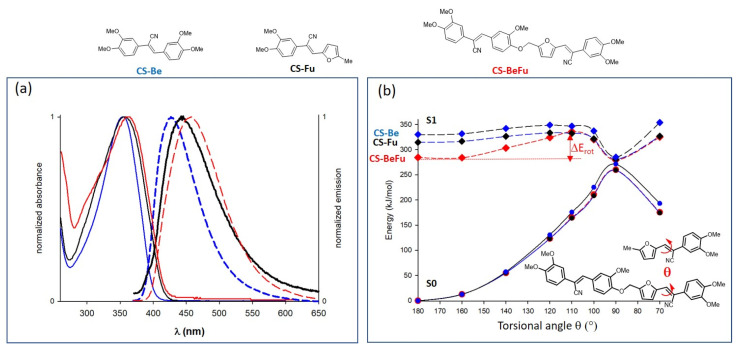
(**a**) Normalized UV–Vis absorption (lines) and emission (dotted lines) in 10^−5^ M solution in cyclohexane of **CS-Be** (blue), **CS-Fu** (black), and **CS-BeFu** (red). (**b**) TD-DFT torsional scans of double bonds θ for **CS-Be** (blue), **CS-Fu** (black), and **CS-BeFu** (red) using the optimized S_0_ state at the TD-B3LYP/6-31G(d,p) level; S_0_ and S_1_ energies were computed for each data point.

The UV–Vis absorption and emission spectra are presented in Figure 3a. The UV–Vis absorption spectra of **CS-Be** and **CS-Fu** present a band around 350 nm corresponding to the HOMO–LUMO transitions with a band gap of about 3.0 eV. The presence of two independent CS units in **CS-BeFu** leads to a larger band and an increase of the molar absorption coefficient. Under excitation at 350 nm, **CS-Be** and **CS-Fu** present a low blue emission in solution in cyclohexane with quantum yields that do not exceed 1%. For the compound **CS-BeFu**, the emission is higher with the quantum yield that reaches Φ_solu_= 15%.

Many cyanostilbene (CS) compounds typically show minimal or no luminescence when dissolved and exposed to irradiation [29]. This fluorescence quenching is often attributed to the steric effect of the cyano group, which triggers the unrestricted rotation of aromatic units around the vinyl group, leading to elevated non-radiative rates. To assess the possibility of luminescence in solution for dicyanostilbene-type derivatives, J. Gierschner et al. propose that the excited Franck–Condon (FC) state energy can evolve with torsion of the double bond to reach the conical intersection (CI) corresponding to a minimum energy of the S_1_ level that is very close to the maximum level reached for S_0_ [41,42,43]. Effective access to the CI, for a 90° torsion of the ethylenic bond, from the FC state energy can be achieved through a rotation barrier (ΔE_rot_). A low ΔE_rot_ value will increase the probability of de-excitation by internal conversion and thus inhibit luminescence. Conversely, an increase in ΔE_rot_ via a stabilisation of the FC state can inhibit effective access to the CI and thus enhance luminescence in solution. To demonstrate access to the CI and calculate ΔE_rot_ values for the **Cs-Be**, **CS-Fu**, and **CS-BeFu** compounds, TD-DFT calculations were performed to estimate the energies of the S_1_ and S_0_ states, following rotation around the cyanovinyl bond with θ angles ranging from 180 to 70° [39]. For the **CS-BeFu** compound, calculations were performed considering the bond of the Fu part. Theoretical calculations were performed considering the B3LYP/6-31G(d,p) basis set and cyclohexane as the solvent. Although TD-DFT is not an appropriate method to describe the region at the CI, TD-DFT is considered to be sufficiently good to describe the path toward the SI [42]. The results for each compound are presented in Figures S3–S5 in the Supplementary Materials. The three compounds show a CI state at θ = 90° with a minimum energy of the S_1_ state close to the maximum energy obtained for the S_0_ state. The access to the CI state from the S_1_ energy at the Franck–Condon state (θ = 180°) can determinate internal conversion possibility, which would be responsible for the non-radiative decay. As shown in Figure 3b, the stabilization of the FC state for the compound **CS-BeFu** leading to a rotation barrier ΔE_rot_ = 39 kJ/mol at the S_1_ state is higher than that for **CS-Fu** and **CS-Be** at 20 kJ/mol. This result reveals that the access to CI is more difficult for **CS-BeFu** and does not allow IC, consequently justifying the fluorescence of **CS-BeFu** in solution.

#### 2.3.2. Emission in THF/Water Mixtures

To study the impact of aggregated states on the emission properties of three compounds, we investigated the fluorescence spectra of various water/tetrahydrofuran (THF) mixtures. THF was chosen for its high miscibility in water, ensuring a homogeneous solution even at high water/THF ratios necessary for aggregating luminogens (see Figure S6 in the Supplementary Materials).

The compounds **CS-Be** and **CS-Fu** exhibit weak emission in pure THF. However, the emission intensity increases as the proportion of water in the solvent reaches 80 vol%, coinciding with the solution becoming slightly cloudy due to aggregate formation. For **CS-Be**, the spectrum of the 95 vol% water solution shows a maximum at 475 nm, corresponding to blue–green emission. This significant increase in emission intensity indicates the formation of strongly luminescent aggregates, demonstrating a pronounced AIE effect. Conversely, **CS-Fu** also exhibits an AIE effect, but it is more moderate, with a less pronounced increase in emission intensity upon aggregate formation. The emission maximum for **CS-Fu** is around 530 nm, indicating weak yellow emission.

For **CS-BeFu**, the emission is more intense, with a maximum at 443 nm. This red shift compared to the emission in cyclohexane is due to the more polar nature of the solvent. Aggregate formation is accompanied by an increase in emission intensity and a red shift of the emission spectrum, reaching a maximum at 524 nm, thus showing more intense yellow emission. This dual emission, blue for the solution and yellow for the aggregates, imparts a Dual-State Emission (DSE) character to the compound.

#### 2.3.3. Emission in the Solid State for the Crystals

**Figure 4:**
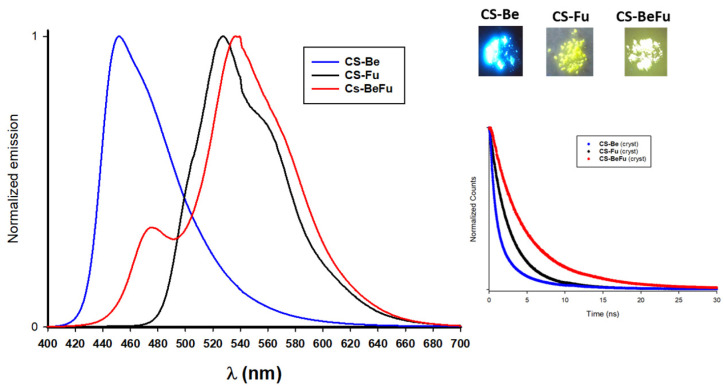
Normalized emission spectra for the crystals of **CS-Be** (blue), **CS-Fu** (black), and **CS-BeFu** (red) under λ_exc_ = 350 nm. Photograph of emission under excitation at 350 nm. Fluorescence lifetime for crystals of **CS-Be** (blue), **CS-Fu** (black), and **CS-BeFu** (red).

As previously demonstrated, the three compounds exhibit varying levels of emission for the aggregates. To facilitate a discussion on the relationship between structure and luminescence properties, photophysical studies were conducted directly on the crystal states (Figure 4). The spectrum of **CS-Be** is characterized by an unstructured band with a maximum at a wavelength of 426 nm and shows a quantum yield of 46%, corresponding to a strong emission in the blue region. The compound **CS-Fu** exhibits more structured emission bands with the appearance of shoulders and a maximum at 527 nm, corresponding to a yellow emission. However, **CS-Fu** is very weakly emissive with a quantum yield of only 5%.

For the compound **CS-BeFu**, which can be considered a combination of **CS-Be** and **CS-Fu**, it is interesting to note that the emission spectrum, characterized by a small band peaking at 476 nm and a more intense band with a maximum at 538 nm, closely resembles that of **CS-Fu** but with a higher quantum yield of 25%, leading to a well perceptible yellow emission. Fluorescence lifetimes for the crystals range from 2.6 ns for **CS-Be** to 5.3 ns for **CS-BeFu**. The electronic properties of the CS compounds being very similar, it is evidently the molecular assembly rather than the molecular structure that can explain the significant differences in the observed photophysical properties of the crystals [1,44,45]. Having the X-ray structures of **CS-Be**, **CS-Fu**, and **CS-BeFu**, it was possible to analyze the structure–property relationships of these three compounds, which present very different emissions for their crystals.

### 2.4. Structure–Property Relationships

**Figure 5:**
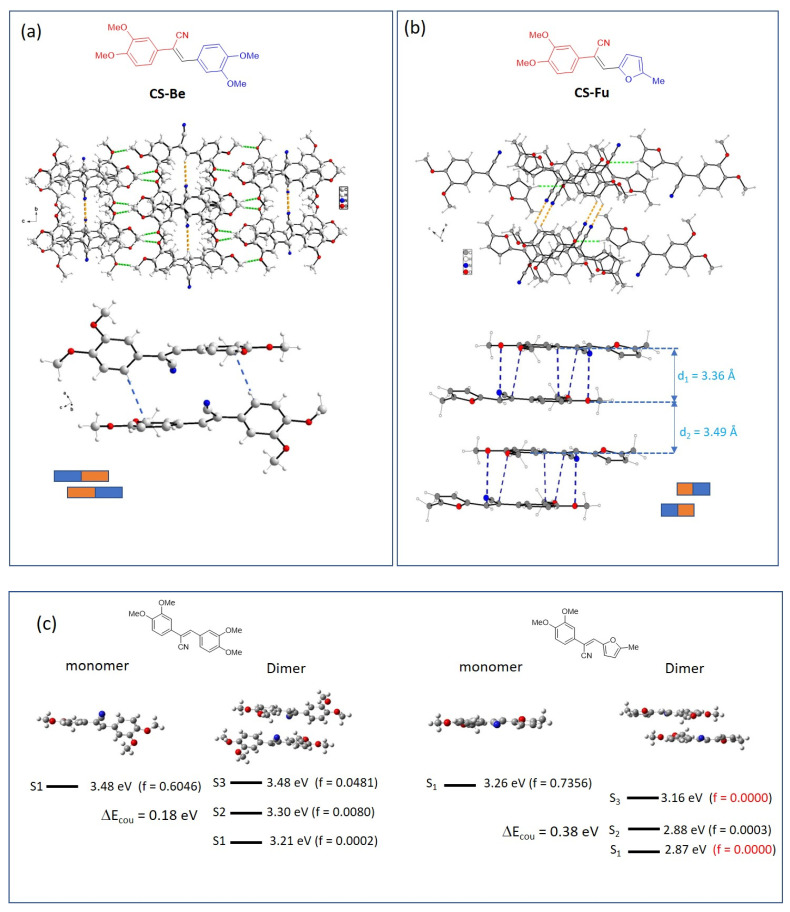
Packing modes of the molecules in crystals. (**a**) **CS-Be**, (**b**) **CS-Fu**. Hydrogen bonds involving the nitrogen atoms are shown as orange dotted lines, those involving the oxygen atoms are shown as green dotted lines, and the short C---C intermolecular distances are shown as blue dotted lines. (**c**) The calculated vertical transition energies of **CS-Be** and **CS-Fu** in the nearest-neighbor configurations according to the single crystal X-ray structures. Energies of the first singlet excited states S1–S3 (oscillator strength values in parentheses).

The structure of **CS-Be** (Figure 5a) exhibits a packing mode characterized by a head-to-tail stacking of molecules along the a-axis, with the dimethoxyphenyl-C=CN unit (in red in Figure 5) overlapping the other dimethoxyphenyl unit. However, due to significant torsion in the molecule, the dihedral angle between the two planes defined by the phenyl rings is 53.5°. There is no π–π stacking, and the shorter interatomic distances between overlapping molecules is d_1_ = 3.48 Å (blue dotted line in Figure 5a). Between adjacent stacking columns, numerous hydrogen bonding interactions occur, involving the nitrogen atoms of the cyano group and the ethylenic hydrogen (highlighted in orange in Figure 5), as well as the oxygen and hydrogen atoms of the methoxy groups (highlighted in green in Figure 5).

For the well different structure of **CS-Fu** (Figure 5b), the nearly planar molecules, featuring a dihedral angle of 20.2° between their two aromatic rings, stack along the a-axis, forming closely contacting dimers. The distances between the planes defined by the phenyl rings and the ethylenic bond are d_1_ = 3.36 Å and d_2_ = 3.49 Å, signifying robust π interactions within **CS-Fu** crystals. These interactions give rise to numerous intermolecular distances with d_C-C_ values less than 3.5 Å.

These significantly different stacking modes account for the substantial variation in emissions between the two compounds. Inter-column interactions through multiple hydrogen bonds in **CS-Be** contribute significantly to limit vibrational and intramolecular motion (RIM) within the solids [3]. The relatively weak π interactions favor each molecule’s contribution as an emissive species with very low excitonic coupling. For compound **CS-Fu**, the formation of dimers with stronger π interactions leads to a significant decrease in luminescence, resulting in a shift towards yellow, indicative of excitonic-type emission. To explore the correlation between the molecular arrangement and the luminescence in the solid state, time-dependent density functional theory (TD-DFT, B3LYP/6-31G(d,p)) calculations were performed with Gaussian 09 by using the classical dimer approach [1]. Monomers and dimers subtracted from each crystal were used for the calculations. The results are presented in Figure 5c. For the **CS-Be** dimer, the TD-DFT calculations give a level S_1_ with a very weak oscillator force *f* than higher levels with more substantial oscillator forces. The excitonic coupling considering the S_2_ level is 0.18 eV. We can therefore consider that in the crystal, it is the monomers that act as the emissive species to dissipate the excitation energy by radiative transitions in the blue. Conversely, for the **CS-Fu** compound, the calculations give null or extremely weak oscillator forces for the first excited states, and the excitonic coupling of 0.38 eV is much greater. This result, which well considers the π–π interactions between the molecules, agrees with the formation of an excimer leading to a weak emission with a shift of the emission into the yellow. The difference between the fluorescence lifetimes for the two crystals also argues for these different types of emission. **CS-Fu** exhibits a longer fluorescence lifetime than **CS-Be**, as expected for an excimer emission in **CS-Fu** [46,47].

**Figure 6:**
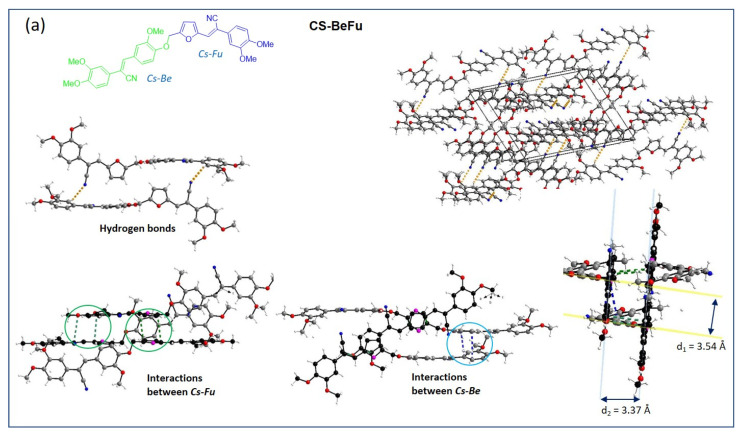

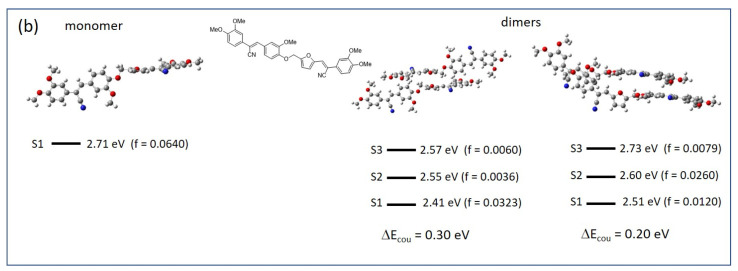
(**a**) X-ray structure of **CS-BeFu** showing the intermolecular interactions. (**b**) The calculated vertical transition energies for the *Cs-Be* and *Cs-Fu* parts in the nearest-neighbor configurations according to the single crystal X-ray structure of **CS-BeFu**. Energies of the first singlet excited states S_1_–S_3_ (oscillator strength values in parentheses).

Considering the X-ray structure of **CS-BeFu** presented in Figure 6a, the two cyanostilbene units, denoted as *Cs-Fu* and *Cs-Be*, are in almost perpendicular planes, and they pile up independently along two directions. The only interactions between the two CS units are via hydrogen bonds between the nitrogen atom of the *Cs-Fu* unit and an aromatic hydrogen atom of the 3,4-dimethoxyphenyl group of the *Cs-Be* unit. The stacking of *Cs-Fu* units is carried out with strong contacts involving furan units (green dotted line in Figure 6) with a distance separating the planes defined by the furans d_2_ = 3.37 Å. However, between the *Cs-Be* units, there are few contacts, and these are weaker; the distance between the planes of the internal phenyl rings is d_1_ = 3.54 Å. For the calculations, both types of dimers were considered (Figure 6b). Interactions between *Cs-Fu* units give excited states with non-zero oscillator strengths and an excitonic coupling of 0.30 eV. Between the *Cs-Be* units, the excitonic coupling of 0.20 eV is weaker. These results are compatible with obtaining a broad band presenting both an emission in the yellow of the excimeric type, which could be due to the *Cs-Fu* part, and another in the blue of the excitonic type for the *Cs-Be* part.

## 3. Experimental Section

### 3.1. Materials and Methods

#### 3.1.1. UV–Vis and Fluorescent Spectroscopy 

Absorption spectra were recorded on a Shimadzu UV-1800 spectrophotometer (Shimadzu, Kyoto, Japan). Fluorescent emission spectra were obtained on an FP-8500 spectrofluorometer (Jasco, Tokyo, Japan). The fluorescence quantum yields both in solution and in the solid state were measured using an ILF-835|100 mm Integrating Sphere (Jasco, Tokyo, Japan).

#### 3.1.2. X-ray Analysis

Crystal data were collected on a Rigaku Oxford SuperNova diffractometer (Rigaku Oxford diffraction, Tokyo, Japan) equipped with an Atlas CCD detector (Rigaku Oxford diffraction, Tokyo, Japan) and micro-focus Cu-K_α_ radiation (λ = 1.54184 Å). The structures were solved by a dual-space algorithm and refined on F^2^ by full matrix least-squares techniques using the SHELX package (G.M. Sheldrick, ShelXT2018/2, ShelXL2018/3-2019/3). All non-hydrogen atoms were refined anisotropically, and the H atoms were included in the calculation without refinement. Multiscan empirical absorption was corrected by using the CrysAlisPro program (CrysAlisPro, Agilent Technologies, Santa Clara, CA, USA, version 1.171.41.118a 2021).

CCDC numbers of all the structures (see Table S1 in the Supplementary Materials) contain the supplementary crystallographic data for this paper. These data are provided free of charge by the joint Cambridge Crystallographic Data Centre and Fachinformationszentrum Karlsruhe Access Structures service (www.ccdc.cam.ac.uk/structures).

### 3.2. Syntheses and Characterization of Compounds

^1^H NMR were obtained at 300 MHz and ^13^C NMR spectra at 75 MHz with CDCl_3_ or DMSO d^6^ as the solvent, and the ^1^H and ^13^C chemical shifts were determined by reference to residual non-deuterated solvent resonances. Data are reported as follows: chemical shift in ppm (δ), multiplicity (s = singlet, d = doublet, t = triplet, m = multiplet), coupling constant (Hz), and integration.

#### 3.2.1. Synthesis of **BeFu**: 5-((4-formyl-2-methoxyphenoxy)methyl)furan-2-carbaldehyde

To a solution of **HMF** (0.5 g, 4 mmol) in 10 mL of DCM, 5 mL of 48% HBr was added. The mixture was stirred at room temperature for 12 h and then washed with a saturated solution of sodium hydrogen carbonate and then with water. After the organic phase was dried and the solvent evaporated, a green oil (0.57 g) was obtained corresponding exclusively to 5-bromomethylfuraldehyde **BrMF**, verified by NMR (yield = 75%).

**BrMF** (0.57 g, 3 mmol) was directly used for the Williamson reaction with 1.2 equivalents of vanillin (0.55 g, 3.6 mmol) and 3 equivalents of K_2_CO_3_ (1.24 g, 9 mmol) in 50 mL of acetone. After refluxing for 12 h, the solid suspension was removed by filtration, and the filtrate was evaporated under vacuum. The residue was purified by chromatography on silica, eluting DCM/AcOEt (8/2) to give the dialdehyde **BeFu** with a yield of 70%.

**BrMF**: ^1^H NMR (CDCl_3_): 9.63 (s, 1H), 7.19 (d, 1H, *J* = 3.6 Hz), 6.58 (d, 1H, *J* = 3.6 Hz), 4.49 (s, 2H).

**BeFu**: ^1^H NMR (CDCl_3_): δ 9.87 (s, 1H), 9.65 (s, 1H), 7.45–7.43 (m, 2H), 7.24 (d, 1H, *J* = 3.6 Hz), 7.05 (d, 1H, *J* = 8.7 Hz), 6.66 (d, 1H, *J* = 3.6 Hz), 5.23 (s, 2H), 3.94 (s, 3H).

#### 3.2.2. Procedure for Knoevenagel Monocondensation

A mixture of aldehyde (4 mmol), acetonitrile derivative (4.4 mmol), and a catalytic amount of ^t^BuOK (0.4 mmol) was added to ethanol without stirring (10 mL) for 6 h at room temperature in the dark. The resulting precipitate was filtered and washed with cold ethanol. Afterwards, it was dried under high vacuum to give the targeted compounds in a crystalline state.

*(Z)-2,3-bis(3,4-dimethoxyphenyl)acrylonitrile* **CS-Be**. Colorless crystalline powders (80% yield). M.p. 155 °C. ^1^H NMR (300 MHz, CDCl_3_) δ 7.67 (d, 2H, *J* = Hz), 7.36 (s, 1H), 7.37–7.34 (dd, *J* = 8.4 Hz, *J* = 2.2 Hz 1H), 7.26–7.22 (dd, *J* = 8.4 Hz, *J* = 2.2 Hz 1H) 7.12 (d, *J* = 2.2 Hz, 1H), 7.13 (d, 2H, *J* = 2.2 Hz), 6.93 (d, *J* = 8.4 Hz, 1H), 6.91 (d, *J* = 8.4 Hz, 1H), 3.97 (s, 3H), 3.96 (s, 3H), 3.95 (s, 3H), 3.93 (s, 3H). ^13^C NMR (75 MHz, CDCl_3_) δ 150.9, 149.8, 149.3, 149.0, 140.5, 127.7, 126.9, 124.0, 118.8, 111.3, 111.0, 110.7, 109.7, 108.7, 108.6, 57.0, 56.0, 55.0. Anal. calcd. (%) for C_19_H_19_NO_4_: C, 70.14; H, 5.89; N, 4.31. Found (%): C, 70.23; H, 5.80; N, 4.45.

*(Z)-2-(3,4-dimethoxyphenyl)-3-(5-methylfuran-2-yl)acrylonitrile* **CS-Fu**. Yellow crystalline powders (65% yield). M.p. 108 °C. ^1^H NMR (300 MHz, CDCl_3_) δ, 7.22–7.19 (dd, *J* = 8.4 Hz, *J* = 2.0 Hz, 1H), 7.20 (s, 1H), 7.09 (d, *J* = 2.2 Hz, 1H), 7.07 (d, *J* = 3.4 Hz, 1H), 6.89 (d, *J* = 8.4 Hz), 6.19 (d, *J* = 3.4 Hz, 1H), 3.94 (s, 3H), 3.91 (s, 3H), 2.40 (s, 3H). ^13^C NMR (75 MHz, CDCl_3_) δ 155.3, 149.6, 149.2, 148.9, 126.8, 126.4, 118.5, 118.2, 116.2, 111.3, 109.5, 109.4, 108.0, 105.3, 56.0, 14.0. Anal. calcd. (%) for C_16_H_15_NO_3_: C, 71.36; H, 5.61; N, 5.20. Found (%): C, 71.44; H, 5.69; N, 5.24.

#### 3.2.3. Synthesis of **CS-BeFu**: (Z)-3-(5-((4-((Z)-2-cyano-2-(3,4-dimethoxyphenyl)vinyl)-2-methoxyphenoxy)methyl)furan-2-yl)-2-(3,4-dimethoxyphenyl)acrylonitrile

To a solution of dialdehyde **BeFu** (520 mg, 2 mmol) in 5 mL of THF at 50 °C, 3,4-dimethoxyphenylacetonitrile **1** (531 mg, 3 mmol) dissolved in 10 mL of ethanol was added and then 20 mg of ^t^BuOK. The solution was stirred at 50 °C for 15 min, and then the mixture was cooled to 0 °C to determine whether precipitation occurred. The solid was filtered, washed with cold ethanol, and then dried under vacuum.

Yellow crystalline powders (45% yield). M.p. 350 °C. ^1^H NMR (300 MHz, DMSO-*d*_6_) δ 7.91 (s, 2H), 7.66 (d, 1H, *J* = 2.1 Hz), 7.58 (dd, 1H, *J* = 8.7 Hz, *J* = 2.1 Hz), 7.39–7.33 (m, 3H), 7.28–7.24 (m, 2H), 7.17 (d, 1H, *J* = 3.6 Hz), 7.09 (dd, 2H, *J* = 8.7 Hz, *J* = 2.1 Hz), 6.93 (d, 1H, *J* = 3.6 Hz), 5.29 (s, 2H), 3.88 (s, 3H), 3.87 (s, 3H), 3.84 (s, 3H), 3.83, and 3.82 (2s, 6H). ^13^C NMR: Solubility too low. HRMS (FAB) *m*/*z* calculated for C_34_H_30_N_2_O_7_Na: 601.1951; found: 601.1955.

## 4. Conclusions

By utilizing biosourced molecules from the lignocellulose platform, such as vanillin and 5-hydroxymethylfurfural, we have developed derivatives from the cyanostilbene family that exhibit specific luminescent properties in both solution and solid states. The Knoevenagel condensation between 3,4-dimethoxyphenyl acetonitrile and either 5-methylfurfural or 3,4-dimethoxybenzaldehyde results in two cyanostilbene units, **CS-Fu** and **CS-Be**, which demonstrate an Aggregation-Induced Emission (AIE) effect with very low emission in solution and significantly higher emission in the solid state. However, when these two CS units are linked without conjugation by a methyleneoxy bridge, creating a nearly perpendicular arrangement of the two conjugated CS arms in the molecule **CS-BeFu**, a luminescent compound with a Dual-State Emission (DSE) effect is obtained, characterized by similar luminescence in both solution and crystalline states. The increase in luminescence in solution is explained by a decrease in the energy of the Franck–Condon excited state for **CS-BeFu** compared to the individual **Cs-Fu** and **Cs-Be** units, which require more energy to overcome the activation energy barrier to reach the energy of the conical intersection of the S_1_ state.

Furthermore, the significantly different emissions in the crystals, both in terms of wavelengths and quantum yields, ranging from intense blue–green for the **CS-Be** compound to low-intensity yellow for **CS-Fu**, with an intermediate yellow–orange emission for **CS-BeFu**, are explained by the considerable differences in the stacking modes of the molecules in the crystals. The weak π interactions observed in the crystals of the **CS-Be** compound induce an excitonic type emission, whereas the strong π interactions in **CS-Fu** favor a much weaker excimeric-type emission that is red-shifted. For **CS-BeFu**, the existence of independent interactions between the two perpendicularly oriented CS arms, with few π interactions for *Cs-Be* and more numerous ones for *Cs-Fu*, results in an emission characterized by a very broad emission spectrum.

## Figures and Tables

**Table 1:** UV–Vis absorption and fluorescence in solution and fluorescence in the solid state.

	CS-Be	CS-Fu	CS-BeFu
Solution ^1^			
λ_abs_ (nm)	352	354	358
ε (mol L^−1^cm^−1^)	23,000	25,600	33,000
ΔE_opt_ (eV) ^2^	3.03	2.95	2.91
λ_em-solu_ (nm)	426	453	457
Φ_solu_ (%) ^3^	<1	<1	15
ΔE_rot_ (kJ/mol) ^4^	19	20	39
Solid ^5^			
λ_em-solid_ (nm)	450	527	476 538
Φ_solid_ (%) ^3^	46	5	25
Δλ_emi_ (eV) ^6^	0.16	0.38	0.41
ΔE_cou_ (eV) ^7^	0.21	0.38	0.20 0.30
τ (ns)	2.3 (85.4%) 4.6 (14.6%)	2.2 (71.0%) 5.1 (29.0%)	1.9 (12.4%) 4.3 (45.7%) 7.2 (41.9%)
τ_aver_ (ns)	2.6	3.1	5.2
k_r_ (ns^−1^) ^8^	0.177	0.016	0.048
k_nr_ (ns^−1^) ^9^	0.21	0.31	0.14

^1^ 10^−5^ M in cyclohexane. ^2^ calculated from the foot of the absorption band. ^3^ Absolute emission quantum yield measured with an integrating sphere. ^4^ Obtained from the TD-DFT calculation. ^5^ Measurements performed for the crystals. ^6^ Δλ_emi_ = λ_em-solu_ − λ_emi-solid_. ^7^ ΔE_cou_ = S_1mono_ − S_1dimer_. ^8^ Radiative rate constants, calculated according to k_r_ = Φ/τ_aver_. ^9^ Non-radiative rate constants, calculated as τ_aver_^−1^ = k_r_ + k_nr_.

## Data Availability

The original contributions presented in the study are included in the article/Supplementary Materials, further inquiries can be directed to the corresponding author.

## Supplementary Materials

The following supporting information can be downloaded at: https://www.mdpi.com/article/10.3390/molecules29133135/s1, Figure S1: ^1^H NMR of compound **CS-BeFU**; Figure S2: Ortep view of **CS-Be**, **CS-Fu** and **CS-BeFu**; Figures S3–S5: TD-DFT torsional scans of double bonds θ for **CS-Be**, **CS-Fu** and **CS-BeFu**; Figure S6: Emission spectra of **CS-Be**, **CS-Fu** and **CS-BeFu** in mixture of THF-Water; Table S1: Crystallographic data of **CS-Be**, **CS-Fu** and **CS-BeFu**.
