# Supplementary material for: Designing Dual-State and Aggregation-Induced Emissive Luminogens from Lignocellulosic Biosourced Molecules
Source: Molecules. 2024 Jul 1;29(13):3135. doi: 10.3390/molecules29133135 (PMC11243483; doi:10.3390/molecules29133135)
Supplement: Supplementary file 1 [file molecules-29-03135-s001.zip › molecules-3061915-supplementary.pdf]

Supporting Information

Designing Dual-State and Aggregation-Induced Emissive Luminogens from Lignocellulosic Biosourced Molecules

Maelys Bonneau, Nagham Ibrahim, Magali Allain, and Pierre Frère
Université d'Angers, CNRS UMR 6200, MOLTECH-Anjou, 2 boulevard Lavoisier, 49045 Angers, France

Figure S1. ^{1}H NMR of compound CS-BeFu 2

Figure S2. Ortep view of **CS-Be**, **CS-Fu** and **CS-BeFu** **3**

Figure S3. TD-DFT torsional scans of double bonds θ for **CS-Be** **4**

Figure S4**.** TD-DFT torsional scans of double bonds θ for **CS-Fu** **5**

Figure S5. TD-DFT torsional scans of double bonds θ for **CS-BeFu** 6

Figure S6. Emission spectra of CS-Be, CS-Fu and CS-BeFu in mixture of THF-Water 7

Table S1. Crystallographic data of **CS-Be, CS-Fu and CS-BeFu** 8

^{1}H and ^{13}C NMR of **CS-Be**, **CS-Fu** and **CS-BeFu** 9 – 13

Mass spectra of **CS-BeFu** 14

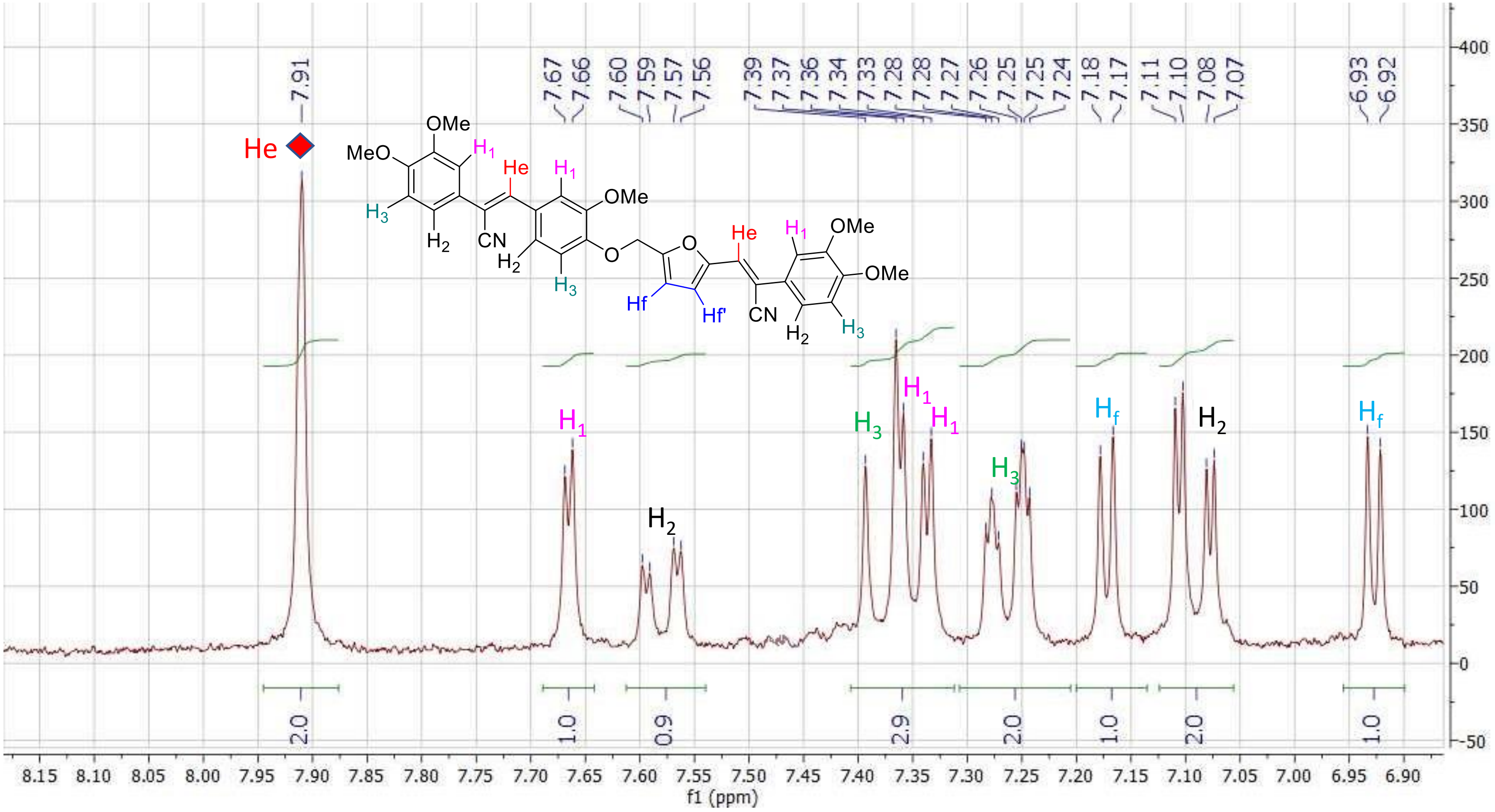

Figure S1: ^{1}H NMR spectra of compound CS-BeFu in DMSO d_6.

CS-Be

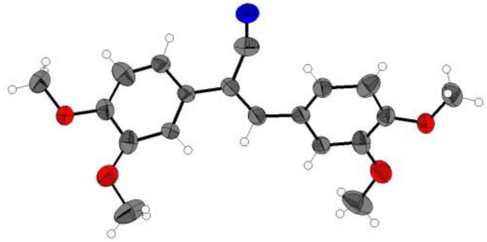

CS-Fu

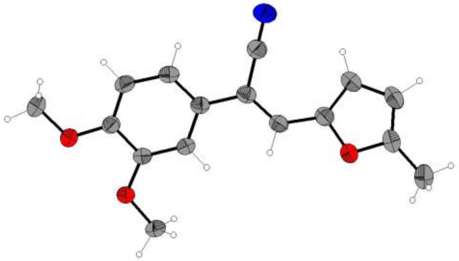

CS-BeFu

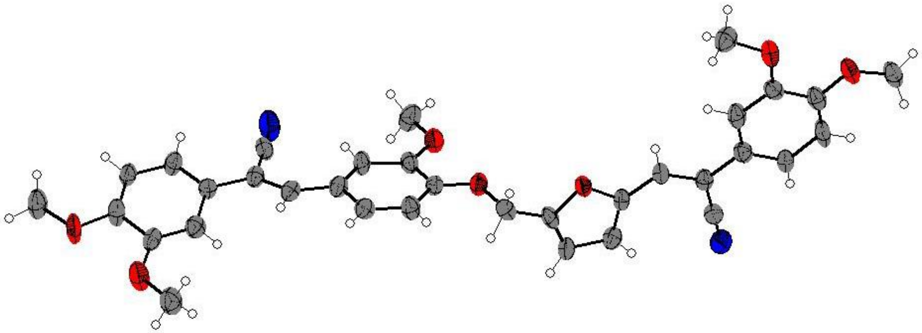

Figure S2: Ortep views of **CS-Be**, **CS-Fu** and **CS-BeFu** showing the Z-configurations of all the cyanovinyl bonds

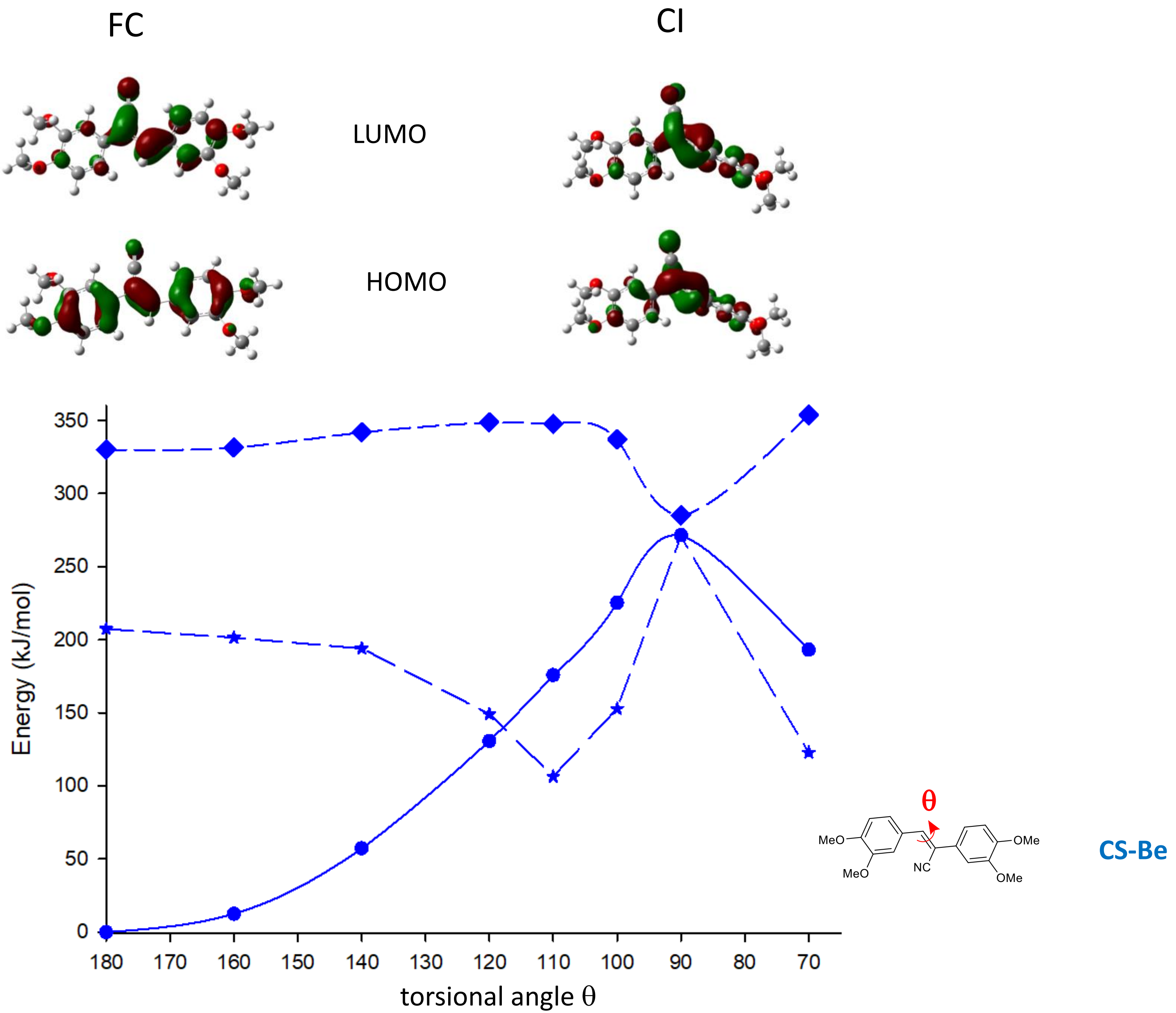

Figure S3 : TD-DFT torsional scans of double bonds θ for **CS-Be**, using the optimized S0 state at the TD-B3LYP/6.31G(d,p) level. S0, S1 and T1 energies were computed for each data point.

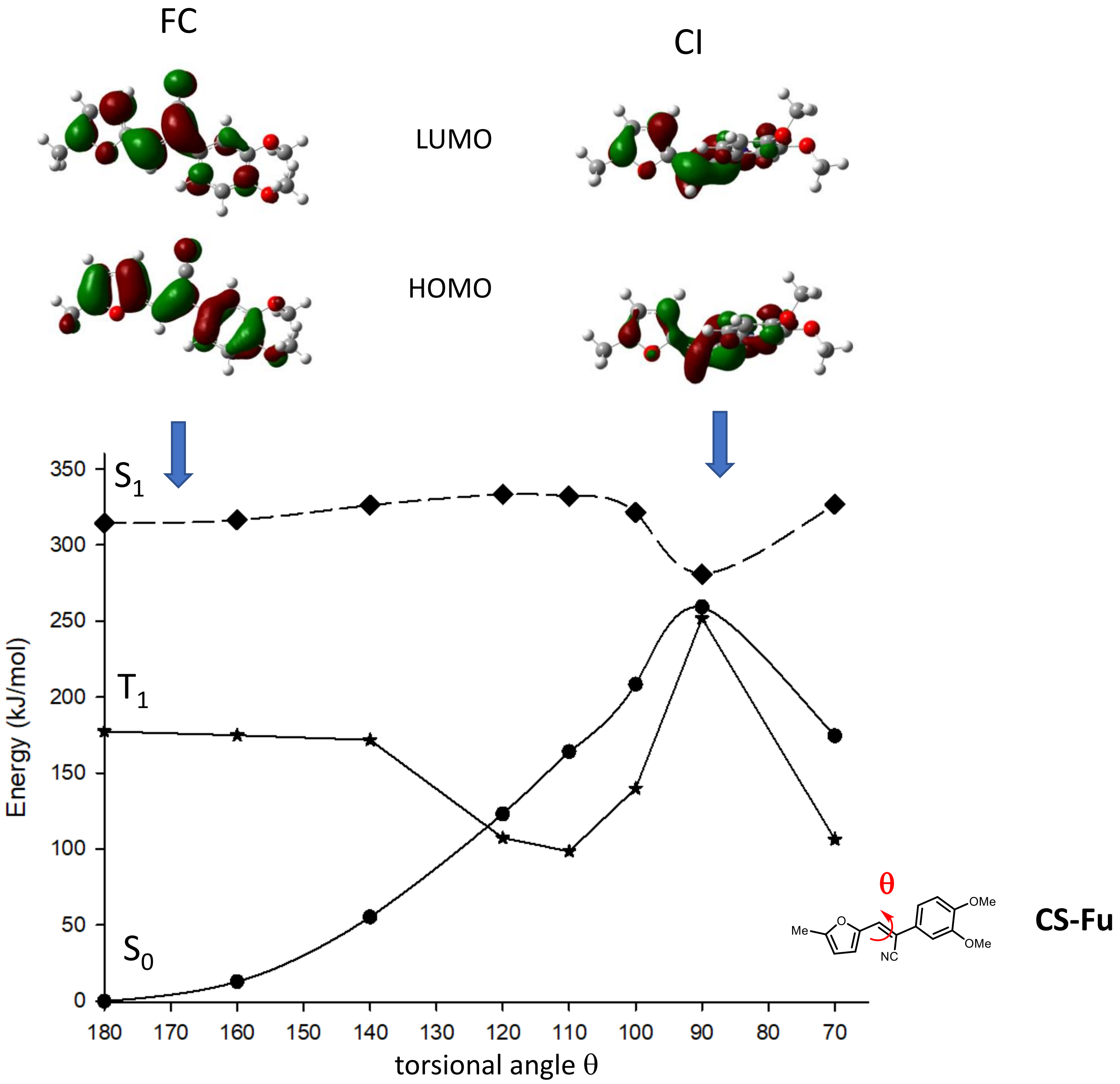

Figure S4 : TD-DFT torsional scans of double bonds q for **CS-Fu**, using the optimized S0 state at the TD-B3LYP/6.31G(d,p) level. S0, S1 and T1 energies were computed for each data point.

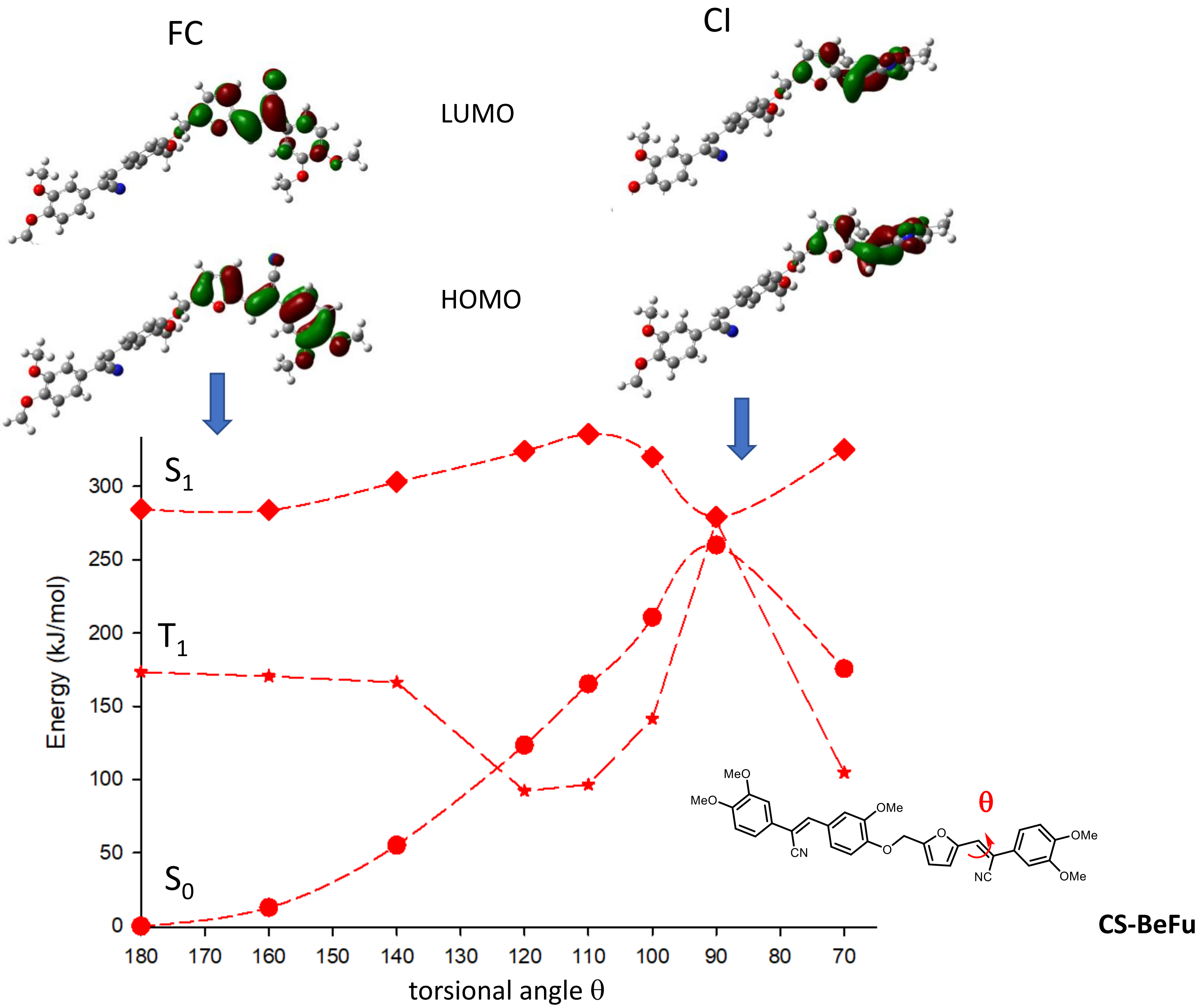

Figure S5 : TD-DFT torsional scans of double bonds θ for **CS-BeFu**, using the optimized S_0 state at the TD-B3LYP/6.31G(d,p) level. S0, S1 and T1 energies were computed for each data point.

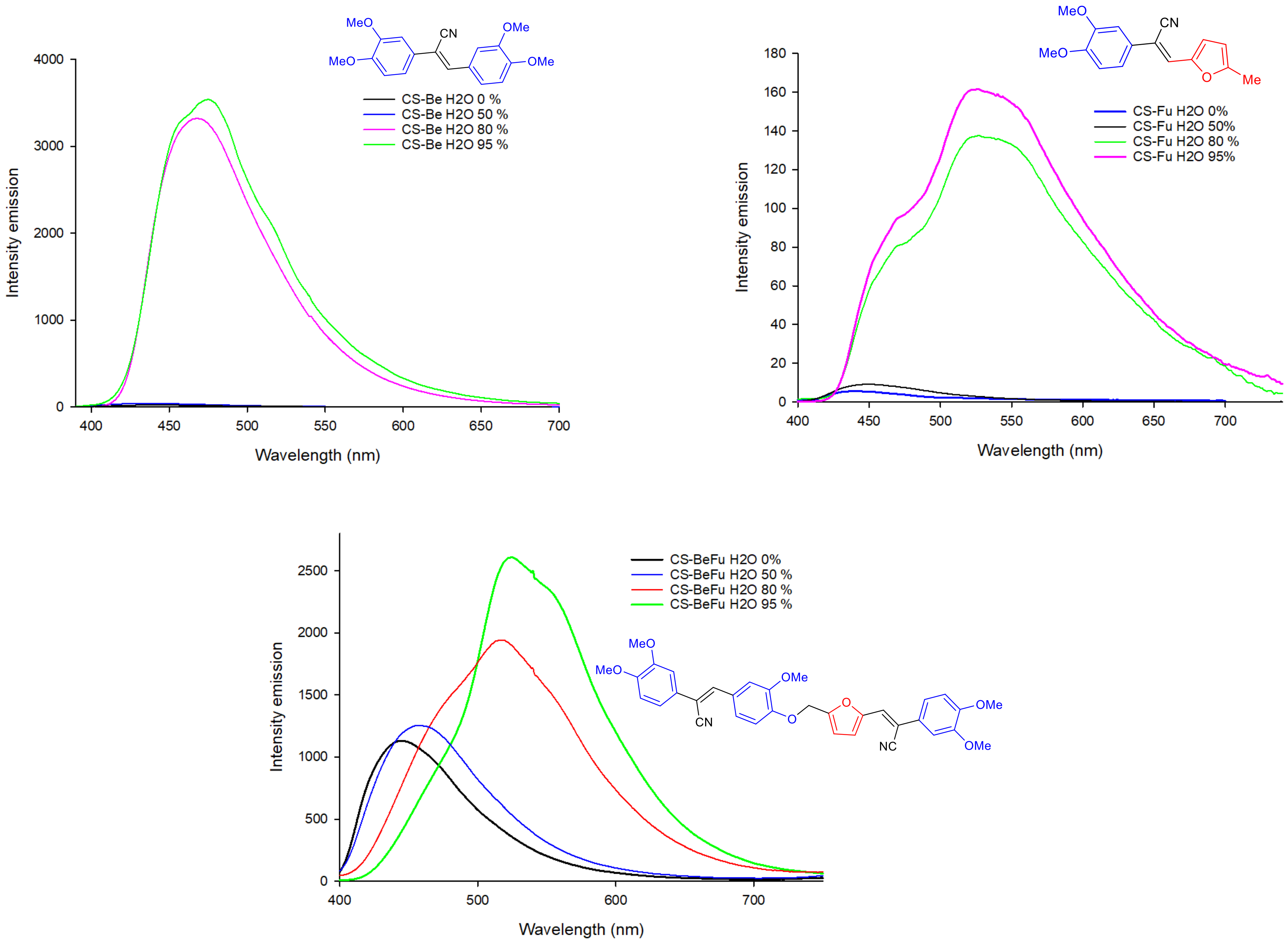

Figure S6, Emission spectra of CS-Be, CS-Fu and CS-BeFu in mixture of THF-Water : THF 100% (black), 50 % vol H2O (blue), 80 % vol H2O (pink) and 95 % vol H2O (green)

Table S1: X-ray crystallographic data of single crystals of **CS-Fu**, **CS-BeFu** and **CS-Be**

	CS-Fu	CS-BeFu	CS-Be
Formula	C16 H15 N O3	C34 H30 N2 O7	C19 H19 N O4
M	269.29	578.60	325.35
T (K)	200.00	200	200
Crystal system<br>Space group	Triclinic,<br>P -1	Triclinic,<br>P 1	Monoclinic,<br>I 2/a
Unit cell.: a (Å)<br>b (Å)<br>c (Å)<br>a (°)<br>b (°)<br>g (°)<br>Volume (Å^3)	6.9278(3)<br>8.9012(4)<br>11.6478(5)<br>71.891(4)<br>85.089(4)<br>82.065(4)<br>675.46(5)	7.1041(3)<br>13.1195(6)<br>17.0173(10)<br>112.145(5)<br>100.909(5)<br>91.569(4)<br>1433.98(13)	9.2182(5)<br>7.4082(4)<br>24.5864(14)<br>90<br>93.688(5)<br>90<br>1675.54(16)
Z	2	2	4
Calculated density	1.324	1.340	1.290
F(000)	284	608	688
θ range (°)	3.997 to 71.889	2.871 to 72.014	3.603 to 75.926
Limiting indices	-8≤h≤7,<br>-9≤k≤10,<br>-26≤l≤24	-8≤h≤8<br>-16≤k≤14<br>-20≤l≤17	-10≤h≤8<br>-7≤k≤9<br>-30≤l≤30
Reflection coll.<br>Unique<br>R (int)	4776<br>2577<br>0.0096	11718<br>6800<br>0.0436	3742<br>1686<br>0.0198
Final R indices, [I>2sigma(I)], R1<br>wR2	0.0357<br>0.1032	0.0461<br>0.1206	0.0433<br>0.114
R indices (all data), R1<br>wR2	0.0368<br>0.1046	0.0587<br>0.1348	0.0495<br>0.1190
Largest diff.<br>Peak and hole (e A^{-3})	0.287<br>-0.283	0.194<br>-0.217	0.144<br>-0.151
CCDC number	2335531	2335530	2335527

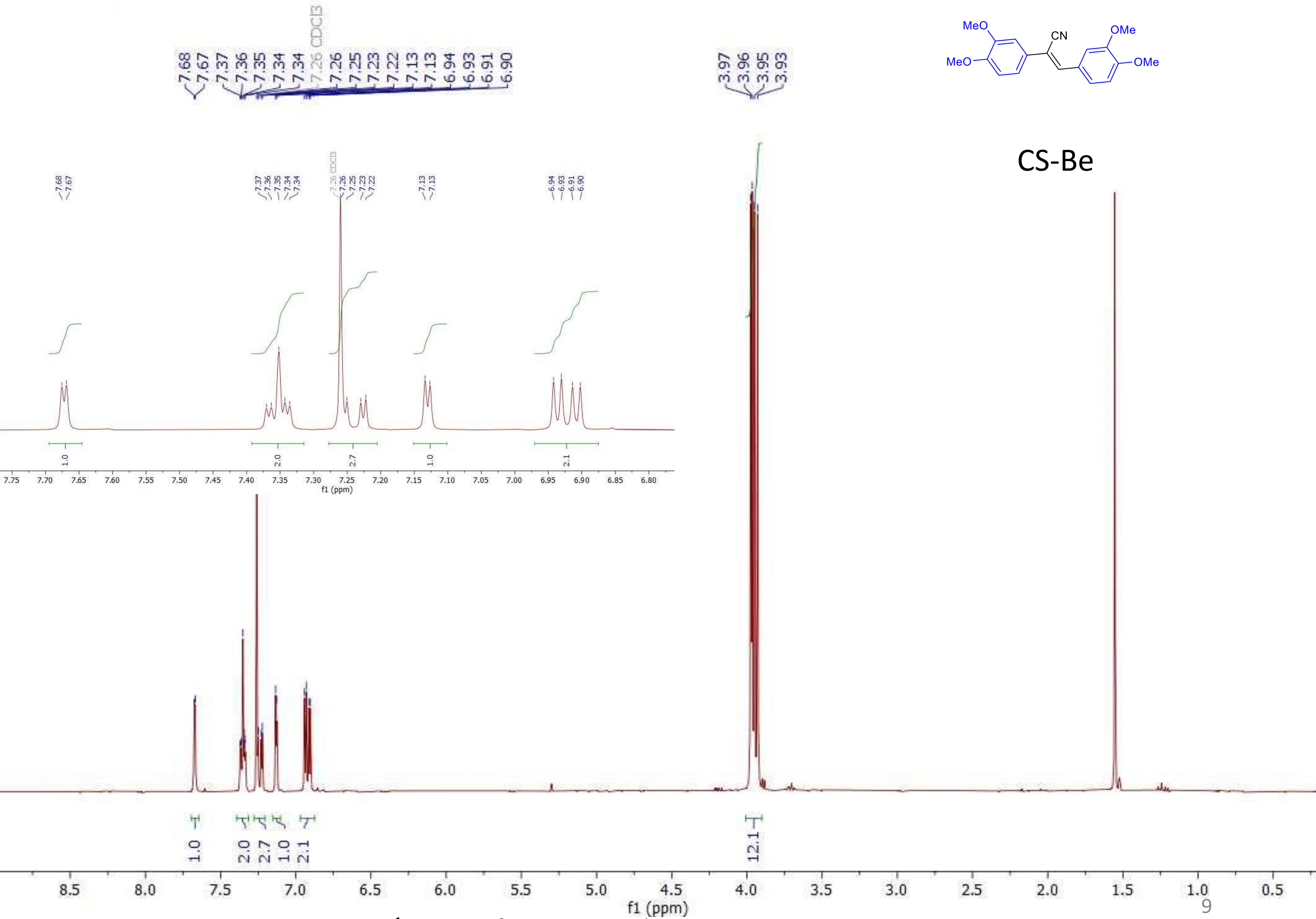

^{1}H NMR of **CS-Be** in $CDCl_3$

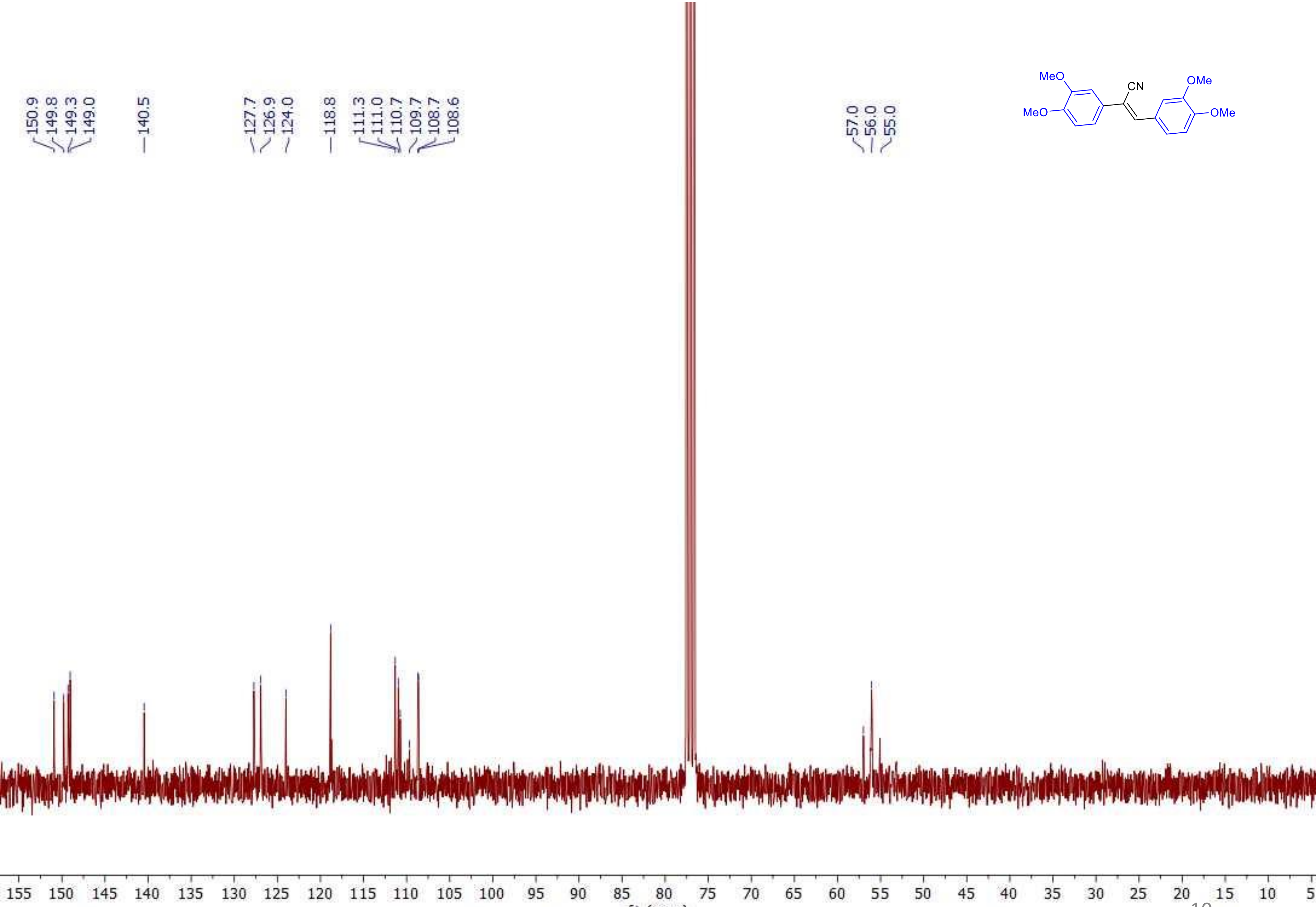

^{13}C NMR of **CS-Be** in $CDCl_3$

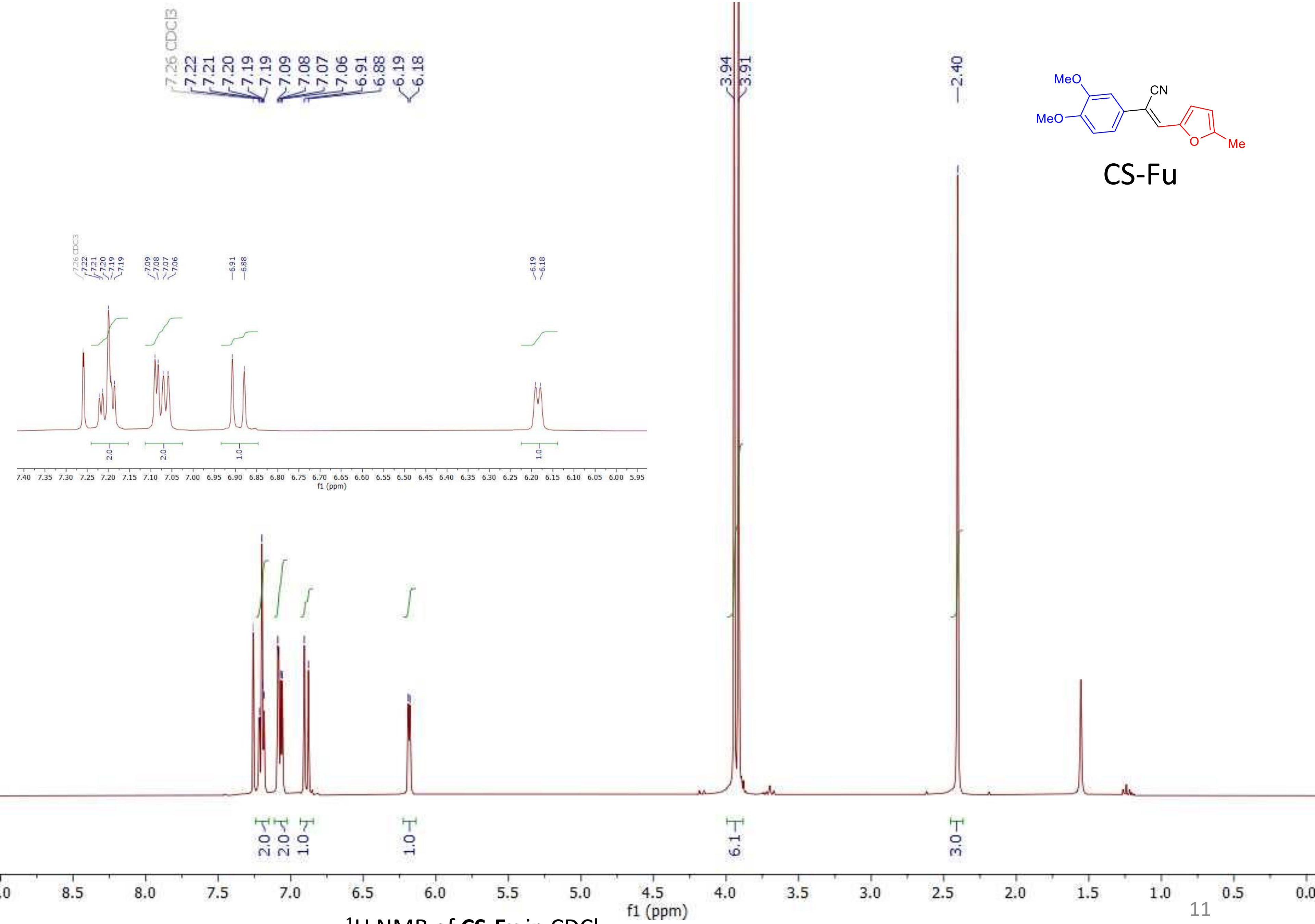

^{1}H NMR of **CS-Fu** in $CDCl_3$

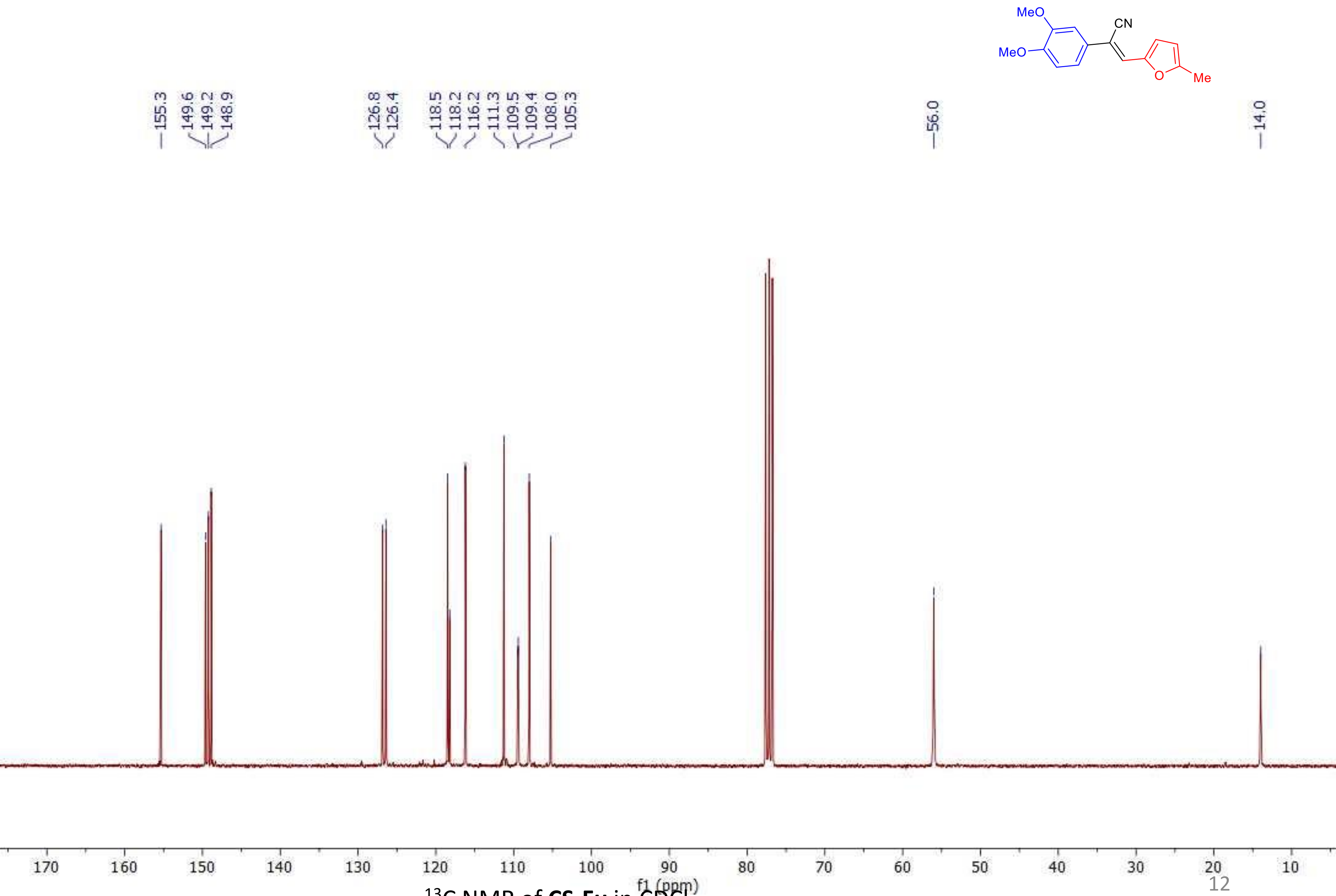

^{13}C NMR of **CS-Fu** in $CDCl_3$

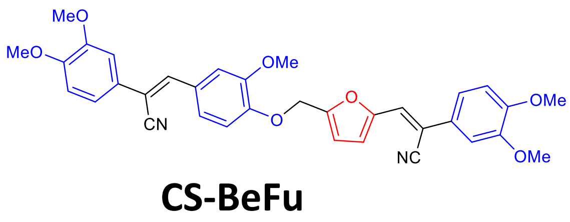

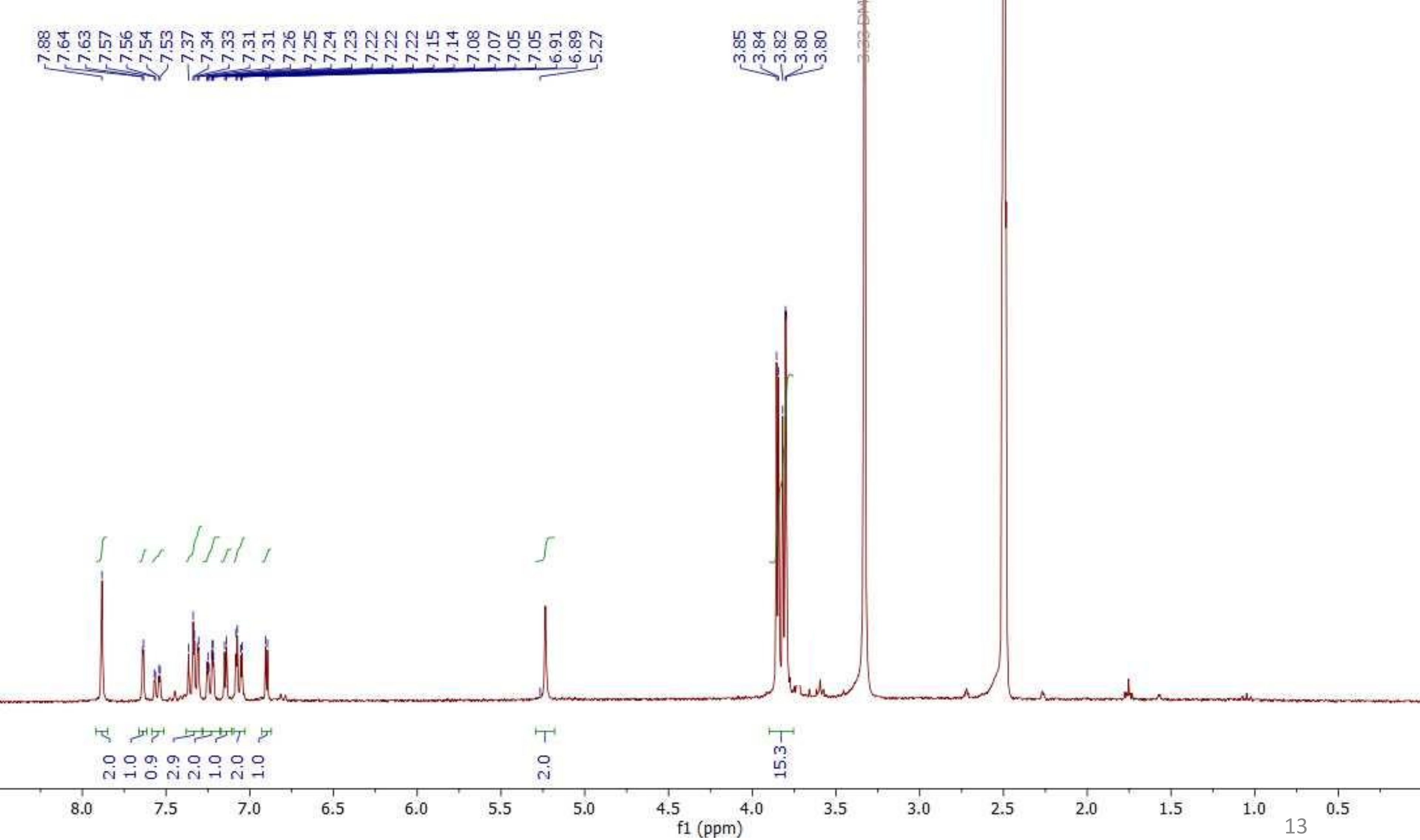

^{1}H NMR spectra of compound **CS-BeFu** in DMSO d_6.

CS-BeFu

RT : 10.74 min Scan# : (2028,2245)
Elements : C 35/0, H 40/0, N 2/0, O 7/0, Na 1/0
Mass Tolerance : 1000ppm, 5mmu if m/z > 5
Unsaturation (U.S.) : -0.5 - 30.0

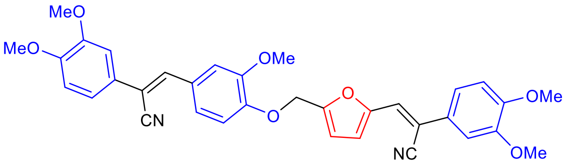

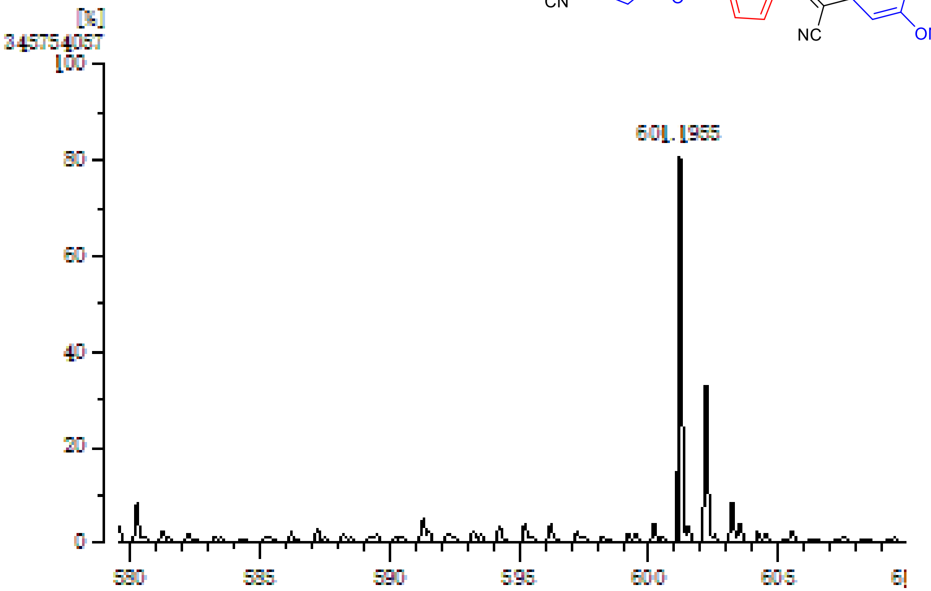

Observed m/z	Int%	Err[ppm / mmu]	U.S.	Composition
1 601.1955	81.15	+0.7 / +0.4	20.5	C34 H30 N2 O7 Na

Mass Spectra (FAB) of **CS-BeFu**
